# Human Herpesvirus 6 Activates NF-κB Signalling and CD163-Positive Macrophage Recruitment in Alcohol-Induced Hepatic Injury

**DOI:** 10.3390/microorganisms13092204

**Published:** 2025-09-20

**Authors:** Anda Upane, Simons Svirskis, Valerija Groma, Sandra Skuja

**Affiliations:** 1 Joint Laboratory of Electron Microscopy, Institute of Anatomy and Anthropology, Rīga Stradiņš University, LV-1010 Riga, Latvia; 044837@rsu.edu.lv (A.U.);; 2 Institute of Microbiology and Virology, Rīga Stradiņš University, LV-1067 Riga, Latvia; simons.svirskis@rsu.lv

**Keywords:** alcohol-induced liver injury, NF-κB signalling, HHV-6 infection, macrophage-specific inflammation-driven CD163 overexpression

## Abstract

Human herpesvirus 6 (HHV-6) establishes lifelong latency in immune cells and may contribute to the progression of ethanol-induced liver injury. To elucidate the contribution of HHV-6 to alcohol-induced hepatic injury, this study evaluated HHV-6 protein expression, NF-κB signalling, and CD163-positive macrophage recruitment in liver samples from control subjects, young individuals with recent alcohol exposure, and individuals with long-term chronic alcohol use. Liver lobules displaying HHV-6 positivity were more frequent in alcohol users (64% in young and 72% in chronic users) compared to controls (48%). CD163-positive macrophage counts were higher in both young and chronic alcohol users compared to controls, with the greatest increase in HHV-6-positive chronic users. NF-κB expression intensity was elevated in alcohol users (*p* < 0.005), and further increased in HHV-6-positive samples (*p* = 0.02). These findings indicate an association between HHV-6 persistence, NF-κB pathway activation, and CD163-positive macrophage-driven inflammatory responses in liver tissue under conditions of chronic alcohol use. Further research is warranted to uncover the mechanisms underlying the interaction between HHV-6 and ethanol in liver injury.

## 1. Introduction

Human herpesvirus 6 (HHV-6), a widely distributed member of the Herpesviridae family, establishes lifelong latency in monocytes and macrophages [1,2]. Alcohol-induced immune system dysfunction—including impaired monocyte and macrophage responses and disrupted cytokine signalling—provides an advantageous environment for HHV-6 persistence and reactivation [3].

Persistent HHV-6 infection has been associated with more severe liver injury, likely through a combination of direct viral effects and immune-mediated responses [4,5]. In cases of acute liver failure—particularly in children—HHV-6 has been frequently detected in liver tissue, often accompanied by histological evidence of immune cell infiltration and hepatocellular damage [4]. Furthermore, improved clinical outcomes with early antiviral intervention were observed in such patients, raising the possibility that viral activity may contribute to disease progression [5]. These findings underscore a potential risk for HHV-6 reactivation in exacerbating liver pathology under conditions of immune dysregulation, such as those seen in chronic alcohol use [6].

This interaction could raise significant concerns in the context of infectious diseases, as it poses a heightened risk to individuals with a history of heavy alcohol use, particularly those with underlying comorbidities or compromised immune function [3,4,5]. Nuclear factor kappa B (NF-κB), a transcription factor that regulates the expression of genes involved in inflammation, apoptosis, and immune responses, plays a central role in mediating hepatic injury [7,8], and HHV-6 has been shown to directly activate NF-κB through viral proteins such as U14, thereby maintaining a pro-inflammatory environment in infected tissues [9]. Recent study has shown that HHV-6B encodes a ribonucleotide reductase that binds the NF-κB p65 subunit, preventing its translocation to the nucleus and thereby attenuating innate immune responses [10]. This ability to both promote and restrict NF-κB signalling under different conditions underscores the virus’s complex strategies for modulating host immunity, which may have important consequences for viral persistence and liver pathology. Cluster of differentiation 163 (CD163), a scavenger receptor primarily expressed on anti-inflammatory macrophages and monocytes [11], reflects anti-inflammatory responses and is often elevated in liver diseases associated with immune modulation and fibrosis, thus suggesting a potential dysregulated response to inflammation and viral persistence, contributing to the pathogenesis of liver injury [12,13]. The aim of this study was to investigate the involvement of HHV-6 in alcohol-induced liver injury by evaluating HHV-6 protein expression, NF-κB immunoreactivity, and CD163-positive macrophage infiltration in liver tissue across different alcohol exposure groups and controls.

## 2. Materials and Methods

### 2.1. Study Groups

Fifty-four liver tissue specimens were obtained from forensic autopsies and divided into three groups: controls (*n* = 11) from young and healthy adults less than 37 years of age, young alcohol users (*n* = 15), also referred to as the age-matched group, and long-term (chronic) alcohol users (*n* = 28). The higher age in the chronic alcohol user group was an intentional aspect of the study design, reflecting the prolonged exposure required for the development of chronic alcohol-related organ pathology. In contrast, the control and age-matched alcohol groups were limited to participants aged ≤37 years, allowing for more appropriate comparison and control of age-related confounding. The collection of tissue samples was monitored using a range of inclusion and exclusion criteria. At autopsy, the criteria for determining the presence or absence of alcohol-related pathology were justified by a macroscopical and microscopical examination of various organs (liver, pancreas, lungs and heart). The alcohol use disorders were confirmed by a certified forensic pathologist. In addition, the criteria used were based on the toxicological examination of blood ethanol ratio and on the compliance of 7–36 h post-mortem interval. Cases not meeting these criteria were excluded and not considered for group allocation.

### 2.2. Immunohistochemistry Procedure and Evaluation of Staining Results

Specimens were stained using the standard haematoxylin and eosin (H&E) procedure and immunohistochemically labelled with a monoclonal mouse antibody against CD163 (1:50; MRQ-26, Cell Marque, Rocklin, CA, USA), a polyclonal rabbit antibody against NF-κB p65 (1:100; ab16502, Abcam, Cambridge, UK), and a mouse monoclonal antibody HHV-6 (20) against viral lysate (1:200; sc-57804, Santa Cruz Biotechnology, Santa Cruz, CA, USA). Primary antibodies were omitted in the negative controls of immunohistochemical reactions. Primary antibody amplification and visualisation were performed using the HiDef Detection HRP Polymer system (Cell Marque, CA, USA) and the 3,3′ diaminobenzidine (DAB) tetrahydrochloride kit (Cell Marque, CA, USA). HHV-6 and CD163 expression were determined quantitatively by counting stained cells in 15 random visual fields for each sample, while NF-κB expression was assessed semi-quantitatively by grading both intensity and distribution on a scale from 0 to 3 through visual scoring in the same 15 visual fields. To provide a more comprehensive evaluation of NF-κB expression across the entire tissue sample, the semi-quantitative scores of all 15 visual fields were summed to obtain an expression score of indicative type, which was included in further analysis. Reactions were evaluated by two independent observers. Microphotographs were obtained as captures using a Glissando Slide Scanner (Objective Imaging Ltd., Cambridge, UK).

### 2.3. Statistical Analysis

Data analysis was conducted using GraphPad Prism version 9.0 (GraphPad Software, San Diego, CA, USA). Power calculations were performed following Cohen’s framework to identify substantial effect magnitudes. Based on Cohen’s sample size methodology, we obtained a sufficiently large effect size (d = 0.7–0.75), and therefore considered that this study, comprising 54 individuals, should provide at least 71–75% power to detect clinically meaningful differences between the study groups. This exceeds the minimum acceptable power of 70%, which is realistic for the effect size to be detected. Summary statistics were generated for participant characteristics and initial measurements. Normality testing of continuous variables was performed using both the D’Agostino–Pearson and Shapiro–Wilk methods. Group comparisons were conducted using the Kruskal–Wallis non-parametric test, with post hoc multiple comparisons adjusted by the Benjamini–Krieger–Yekutieli (BKY) two-stage linear step-up procedure to control the false discovery rate. Fisher’s (F) exact test was used to analyse categorical data. Results are presented as median values with interquartile range (IQR) as a variance factor. A significance threshold of a *p*-value less than 0.05 is to determine statistical significance.

## 3. Results

### 3.1. Characteristics of Study Groups

Three study groups were established according to the specified criteria: young and healthy individuals (non-alcoholic control group; median age 32 years (IQR 22–36), age range 17–37 years), age-matched alcohol users (young adult alcohol dependents; median age 31 years (IQR 26–34), age range 22–35 years), and chronic severe alcohol users (median age 52 years (IQR 41–60), age range 31–66 years). The basic characteristics of these groups are summarised in Table 1.

### 3.2. Comparative Analysis of CD163-Positive Macrophage Density in HHV-6-Positive Liver Lobules of Control and Alcohol-Exposed Individuals

**Figure 1:**
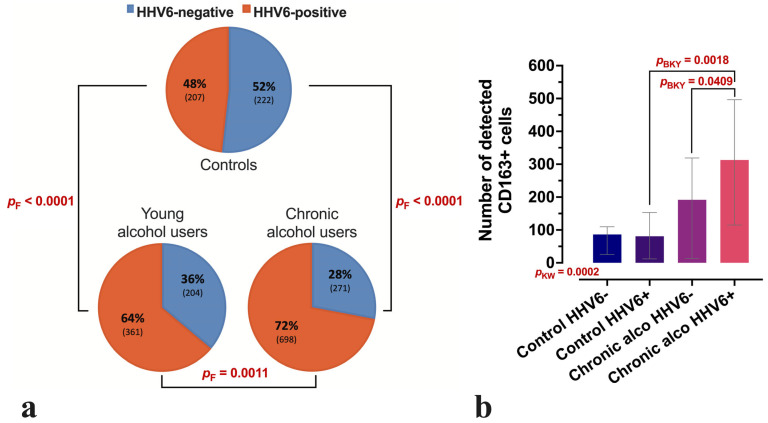
Distribution of liver lobules displaying HHV-6 positivity and CD163-positive cells across the study groups. (**a**) Proportional distribution of HHV-6-positive and HHV-6-negative liver lobules in control, young alcohol user, and long-term (chronic) alcohol user groups, providing a comparative visualisation of HHV-6 positivity across all study groups. The corresponding number of lobules is given in parentheses. (**b**) CD163-positive cell counts in the control group and chronic alcohol user (chronic alco) group, with a distinction between HHV-6-positive and negative samples. Between-group comparisons were performed using Fisher’s (F) exact test (**a**) or the non-parametric Kruskal–Wallis (KW) test with the Benjamini–Krieger–Yekutieli (BKY) two-stage linear step-up procedure as the post hoc adjustment (**b**). Boxes represent the median with interquartile range (IQR) (**b**).

Liver lobules exhibiting HHV-6 positivity were identified in 48% of controls, 64% of young alcohol users, and 72% of chronic alcohol users (Figure 1a). The median number of CD163-positive cells in the lobular area was increased in young alcohol users (median = 135, IQR 8–218), and to a greater extent in chronic alcohol users (median = 216, IQR 40–330), compared with controls (median = 56, IQR 7–100). The most pronounced increase was observed in HHV-6-positive chronic alcohol users (median = 313, IQR 115–496). The control and chronic alcohol user groups, both HHV-6-positive and HHV-6-negative individuals, are presented in Figure 1b to illustrate differences in CD163-positive cell counts.

**Figure 2:**
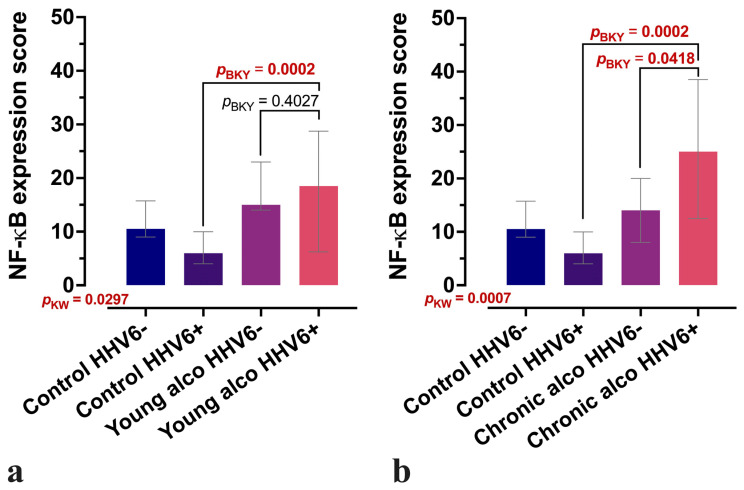
NF-κB expression in liver tissue samples with and without confirmed HHV-6 positivity. (**a**) Changes in NF-κB expression scores in the young alcohol user (young alco) group compared to controls, stratified by confirmed HHV-6 positivity. (**b**) Changes in NF-κB expression scores in chronic alcohol user (chronic alco) group compared to controls, stratified by confirmed HHV-6 positivity. The *Y*-axis shows the indicative-type score of NF-κB expression, where the semi-quantitative scores of all 15 visual fields are summed across the entire tissue sample. Between-group comparisons were performed using non-parametric Kruskal–Wallis (KW) test with the Benjamini–Krieger–Yekutieli (BKY) two-stage linear step-up procedure as the post hoc adjustment. Boxes represent the median with interquartile range (IQR).

**Figure 3:**
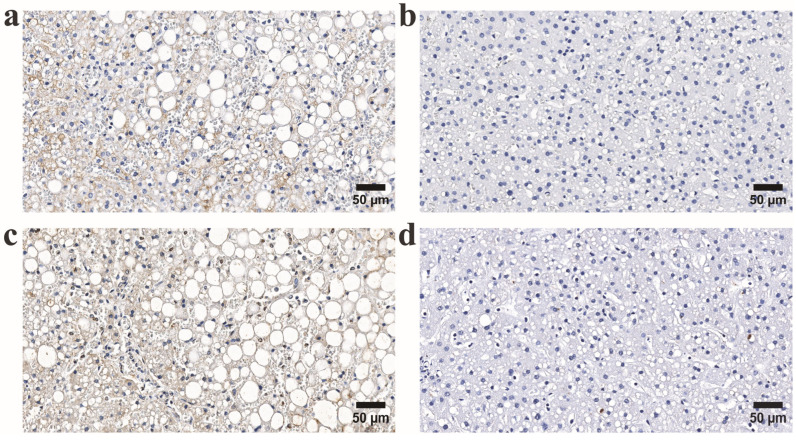
Representative immunohistochemical micrographs of liver tissue from individuals in the long-term alcohol use group. (**a**,**b**) Upper panel: immunohistochemical staining for HHV-6 in HHV-6-positive (**left**) and HHV-6-negative (**right**) cases. (**c**,**d**) Lower panel: corresponding NF-κB expression in the same tissue regions. Notably, HHV-6-positive samples exhibit increased NF-κB immunoreactivity relative to HHV-6-negative counterparts.

The intensity of NF-κB expression in the lobular area was significantly higher in young alcohol users (*p* < 0.005). In addition, both intensity and distribution were notably increased in chronic alcohol users (*p* < 0.001, *p* = 0.02) compared to controls. Dividing the study groups into HHV-6-positive and HHV-6 negative samples revealed a significant increase in the intensity of the NF-κB immunoexpression in HHV-6-positive samples from both young alcohol user and chronic alcohol user groups (Figure 2a,b and Figure 3).

**Figure 4:**
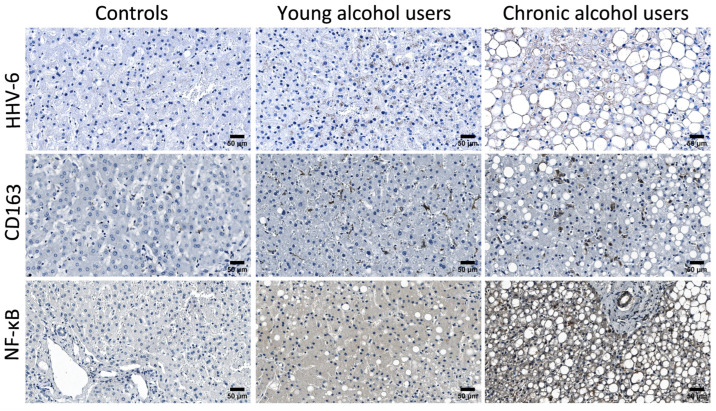
Representative microphotographs of liver tissue immunolabelled using anti-HHV-6, anti-CD163, and anti-NF-κB antibodies in all three analysed study groups—control group, young alcohol user group and chronic alcohol user group. A panel of the 1st row shows increasing HHV-6 expression as well as progressive tissue injury across study groups. A panel of the 2nd row shows a few CD163-positive cells in the control group, with increasing counts in both young alcohol user and chronic alcohol user groups, accompanied by progressive liver tissue injury across study groups. A panel of the 3rd row shows the increasing intensity of NF-κB immunoexpression across all study groups, with similar distribution patterns and progressive liver tissue injury.

The observed immunohistochemical patterns and morphological changes reflect the progression of liver tissue injury across the study groups with preserved hepatic architecture in the control group (Figure 4). Typical features of chronic alcohol-induced liver injury, including macrovesicular steatosis, fibrosis, inflammatory infiltration, and consequent hepatocyte loss, become more prominent with sustained ethanol exposure. Our results demonstrate a high number of microvesicular lipid inclusions in alcoholic individuals. Both microvesicular and macrovesicular steatosis, along with ballooned hepatocytes, were observed in young alcohol users (Figure 4, middle column). In chronic alcohol users, fibrosis was frequently present, often accompanied by portal tract inflammation. Additionally, islands of hepatocytes, with nodules completely surrounded by connective tissue septa were commonly observed (Figure 4, right column).

## 4. Discussion

Our findings support the idea that chronic alcohol consumption impairs immune function, which may promote reactivation of latent viruses such as HHV-6 and contribute to liver inflammation and tissue injury. Alcohol is known to dysregulate both innate and adaptive immunity, particularly through its effects on monocytes and macrophages, creating an opportunity for viral reactivation [1,3,5]. Reactivation of HHV-6 in immunocompromised individuals, including transplant recipients and those with alcohol-induced immunosuppression, has been associated with adverse outcomes [2,4,6]. Recent epidemiological data indicate that alcohol consumption significantly increases the risk of herpesvirus reactivation, for example, individuals with a history of alcohol use show up to a ~1.8-fold higher odds of HSV-2 infection compared to abstainers [14]. Given that HSV-2 and HHV-6 both belong to the Herpesviridae family and share the ability to establish latency and reactivate under immunosuppressive conditions, similar mechanisms may contribute to HHV-6 reactivation in individuals with chronic alcohol consumption.

Moreover, HHV-6 has been increasingly recognised for its clinical implications in liver disease. It has been associated with autoimmune hepatitis [4,15], and case reports have drawn parallels with immune-triggered hepatic damage seen in hepatitis A and B virus infections [16,17,18]. In transplant medicine, active HHV-6 replication has been observed in hepatocytes and bile duct cells using molecular methods, and its reactivation has been implicated in liver graft dysfunction and failure [4,6,19]. A notable study found HHV-6 antigens in 80% of explanted livers from patients with unexplained acute liver failure, strongly implicating the virus in severe hepatic compromise [20].

Our observation of increased HHV-6-positive cells in liver tissue among chronic alcohol users aligns with these findings and indicates a possible association between alcohol use and viral reactivation. We also observed significantly elevated NF-κB expression in alcohol-exposed liver tissue, supporting its role as a central mediator of inflammation. HHV-6A tegument protein U14 has been shown to directly activate NF-κB [9], and alcohol independently affects this pathway in liver disease [8], suggesting a synergistic inflammatory effect. Alcohol consumption is a well-established activator of NF-κB signalling, and our findings are in line with this concept [8]. However, the role of NF-κB in liver disease is multifaceted and context dependent. In this regard, our observation that NF-κB immunostaining is particularly enhanced in HHV-6-positive alcoholic livers (Figure 2) provides an additional layer of complexity. Although these data do not establish a direct mechanistic role, they point to a potential interaction between viral co-infection and inflammatory signalling cascades, suggesting that HHV-6 may amplify NF-κB-mediated responses in the alcoholic liver microenvironment. Indeed, viruses are known to employ diverse strategies to manipulate NF-κB pathways, activating pro-inflammatory cytokine production while simultaneously encoding inhibitors of key signalling components to promote immune evasion [21,22]. At the same time, viruses can exploit monocytes/macrophages for dissemination and persistence [23], which further underlines the relevance of our observations. While our findings do not establish causality, they demonstrate a significant association between HHV-6 infection and enhanced NF-κB immunostaining in alcoholic livers, providing a basis for future, more detailed mechanistic investigations. While our current findings provide important insights into the potential relationship between HHV-6 and alcohol consumption, future studies are needed to distinguish the specific contributions of HHV-6A and HHV-6B. Clarifying which variant plays a more significant role in liver pathology would enhance our understanding of virus-alcohol interactions and potentially inform targeted diagnostic or therapeutic approaches.

The increase in CD163-positive cells in liver tissue from young and chronic alcohol users is consistent with findings that link CD163 expression to liver inflammation, fibrosis, and viral immune response modulation [11,13]. Our data suggest that hepatic macrophages undergo significant phenotypic remodelling in alcohol-related liver injury. However, despite the use of multiple markers, our data reaffirm the limitations of current immunohistochemical approaches in definitively distinguishing between resident Kupffer cells and recruited monocyte-derived macrophages within formalin-fixed, paraffin-embedded tissue. Accordingly, while our observations clearly document a heightened presence of CD163-positive macrophages in the alcoholic liver, we have refrained from assigning definitive lineage origin to CD163-positive cells in the present study. This decision reflects the current methodological constraints and the need for further studies to resolve macrophage ontogeny in situ.

This study has several limitations that should be acknowledged. First, the liver tissue specimens were obtained post-mortem from forensic autopsies, which, while allowing access to otherwise unavailable material, may not fully reflect the dynamic processes occurring in living subjects. Second, due to the post-mortem origin of the liver tissue samples, clinical data regarding alcohol consumption patterns, duration of alcohol use, comorbid medical conditions, or potential co-infections were not available, thereby limiting the ability to correlate histological findings with detailed clinical backgrounds. Third, the sample size was limited, particularly within the control and age-matched alcohol user groups, which may affect the statistical power and generalisability of the findings. Lastly, the detection of HHV-6, NF-κB activation, and CD163 expression was based on immunohistochemical methods, which are semi-quantitative and may be influenced by tissue preservation and staining variability. Despite these limitations, the results provide novel insight into virus-associated immune responses in alcohol-related liver injury.

## 5. Conclusions

In this study, we observed an increased number of HHV-6-positive cells alongside elevated NF-κB activation and CD163 expression in liver tissue from alcohol users compared to controls. Further studies are needed to confirm these interactions and explore the mechanisms potentially linking HHV-6 persistence and ethanol-induced liver tissue injury.

## Figures and Tables

**Table 1:** Key characteristics of the study groups, including number of individuals, sex distribution, age, and blood ethanol concentration in the study population.

Group	*n*	Female	Male	Medan Age (IQR)	Blood Ethanol (‰)
Controls	11	1	10	32 (22–36)	0
Age-matched alcohol users	15	2	13	31 (26–34)	0.59–4.61
Chronic alcohol users	28	8	20	50 (41–60)	1.07–8.44

## Data Availability

The original contributions presented in this study are included in the article. Further inquiries can be directed to the corresponding author.
